# Anti-VEGF and anti-EGFR agents open up new horizons changing the landscape for their associations

**DOI:** 10.1007/s00280-016-3081-z

**Published:** 2016-06-16

**Authors:** Gerard Milano

**Affiliations:** Nice, France

To the Editor

Zaniboni and Formica recently published a review article considering preclinical and clinical data in the current context of optimal therapeutic sequences in metastatic colorectal cancer [1]. Their conclusions supported anti-EGFR agents as first-line treatment before anti-VEGF agents. Different biological and molecular treatment-conferred changes were advocated to support this order of drug combination. Surprisingly, an important aspect which was not considered by the authors is the current reconsideration of anti-VEGF and anti-EGFR effects through their respective and complementary abilities to modulate tumor immunity both directly and indirectly. For instance, on the one hand, involvement of T cells has been reported to be a component of the antitumor activity of EGFR-targeted monoclonal antibodies [2]. Also, EGFR activation has recently been shown to upregulate PDL-1 [3], potentially implying that EGFR inhibition can restore anti-tumor immunity. On the other hand, apart from its pro-angiogenic effect, VEGF must also be considered to be a strong immunosuppressor agent [4] and VEGF-targeted therapy has been shown very recently to combine favorably with checkpoint inhibitors based on pronounced CD8^+^ T cells tumor infiltration boosted by the anti-angiogenic drug [5].

Taken all together, these different features must lead us to reconsider anti-VEGF and anti-EGFR combinations, focusing not only on the association but also on the prospect of perspective for combinations with immunomodulatory agents like checkpoint inhibitors.
